# INSM1, a Novel Biomarker for Detection of Neuroendocrine Neoplasms: Cytopathologists’ View

**DOI:** 10.3390/diagnostics11122172

**Published:** 2021-11-23

**Authors:** Zahra Maleki, Akash Nadella, Mohnish Nadella, Gopi Patel, Shivni Patel, Ivana Kholová

**Affiliations:** 1 Division of Cytopathology, Department of Pathology, The Johns Hopkins Hospital, The Johns Hopkins Medical Institution, Baltimore, MD 21218, USA; 2 Department of Pathology, Johns Hopkins University, Baltimore, MD 21218, USA; anadell1@jhu.edu (A.N.); mnadell3@jhu.edu (M.N.); gpatel6@jhu.edu (G.P.); spate159@jhu.edu (S.P.); 3 Department of Pathology, Fimlab Laboratories, Tampere, Faculty of Medicine and Health Technology, Tampere University, 33100 Tampere, Finland; ivana.kholova@sll.fimnet.fi

**Keywords:** cytology, neuroendocrine tumor, INSM-1 immunostaining, medullary thyroid carcinoma, pancreatic neuroendocrine tumor, pulmonary neuroendocrine neoplasms, carcinoid

## Abstract

Background: Insulinoma-associated protein 1 (INSM1) has been considered as a novel immunostain for neuroendocrine tumors (NETs) and is hypothesized to be more reliable than first-generation NET biomarkers, such as CGA (chromogranin A), SYP (synaptophysin) and CD56 (neural cell adhesion molecule). In this review, we summarize existing literature on INSM1′s reliability as an immunostain for detection of various NETs, its results in comparison to first-generation NET biomarkers, and its expression in both non-NETs and benign tissues/cells on cytology specimens (cell blocks/smears).

**Summary:** INSM-1 immunostain has been utilized in diagnosis of NETs with variable tumor differentiation from different body sites on cytology specimens. The nuclear expression of INSM-1 is more pronounced in less differentiated NETs on cytologic material regardless of the body site. In the head and neck area, sinonasal neuroendocrine carcinoma, neuroendocrine carcinomas localized in the hypopharynx larynx trachea and parapharyngeal space as well as HPV-related neuroendocrine carcinomas, paraganglioma and medullary thyroid carcinoma, are shown to be immunoreactive for INSM1. Thyroid teratoma with recurrent *DICER1* hotspot mutations showed patchy INSM1 positivity and parathyroid cells were negative for INSM1. Pulmonary NETs, including small cell lung carcinoma, large cell NE carcinoma, atypical carcinoid tumor and typical carcinoid tumor, expressed INSM1 with high specificity (97%) that was similar to CGA (98%) but greater than CD56 (87%) and SYP (90%). INSM1 displayed 100% sensitivity, specificity, PPV, and NPV for differentiating pancreatic NETs from non-NE pancreatic tumors. SPN, ACC, and PDAC were always INSM1 negative (unlike SYP which was positive for SPN) on cell block samples making INSM1 a useful marker for excluding possible non-NETs. INSM1 has also been shown to be more sensitive (92.3%) than both SYP (84.6%) and CGA (53.8%) in prostatic small cell carcinomas, making it a superior NE biomarker. Moreover, it is highly specific considering that only 3.4% of benign prostate tissues and 4.0% of prostate adenocarcinomas stained positive for INSM1. Among non-neoplastic tissues, normal cortical adrenal cells and a small percentage of prostate cells were shown to be positive for INSM1.

**Key messages:** INSM-1 nuclear expression in cytology specimens successfully shows that it is a robust neuroendocrine marker. To conclude; numerous studies confirm that INSM1 is a reliable immunostain for the characterization of NETs with high sensitivity and specificity.

## 1. Introduction

In 1992, a subtraction library was constructed from rare human insulinoma and glucagonoma tissues [1] to unveil the specific antigens associated with insulinoma and/or antigens that encode autoantigens in type 1 diabetes. The clones isolated from this subtraction library were named insulinoma-associated-1 (IA-1) or insulinoma-associated-2 (IA-2) among others. IA-1 was the very first antigen identified by the subtraction library. The IA-1 gene was later renamed in the GenBank database as INSM1 (insulinoma-associated-1). Analysis of INSM1 primary structure revealed the presence of 5 Cys2 His2 type zinc finger DNA binding motifs in the C terminus, categorizing the protein as a zinc finger DNA binding protein [2].

INSM1 is a protein that plays a cardinal role as a transcription factor in the differentiation of embryonic neuroendocrine cells. In situ hybridization studies of human tissues have shown the presence of the INSM1 transcript across various brain regions, as well as in endocrine organs such as the pancreas, adrenal gland, thymus, thyroid, and the endocrine cells of the gastrointestinal tract [3]. Though INSM1 expression normally decreases drastically following birth and is nearly gone by adulthood, INSM1 has been shown to be transiently expressed in both neuronal progenitor cells and nascent neurons [4,5]. Numerous studies have demonstrated how INSM1 plays a crucial role in neuroendocrine differentiation in both neoplastic and normal pancreatic endocrine tissues. Overexpression of the islet-specific transcription factors (ITFs) INSM1, Pdx-1, and NeuroD1 in the AR42J pancreatic tumor cell line results in higher levels of differentiation into insulin-positive cells and the activation of Ngn3 and MafA [6]. INSM1 was the ITF shown to be directly responsible in coordinating this conversion into insulin-positive cells, while the addition of the other two ITFs simply increased the efficiency of this trans-differentiation [6]. These results were also replicated when tested in the Panc-1 (ATCC) cell line [7]. INSM1 null cells show lower rates of proliferation, with a range of characteristics implicating cell cycle arrest at the G1/G2 phase rather than increased apoptosis [8]. On the other hand, increases in proliferation, found in endocrine cells differentiated in the presence of INSM1 are associated with changes in the activity of cell cycle inhibitors Ripply3 and Cdkn1b [9]. INSM1-dependent endocrine differentiation also causes the expression of key extracellular matrix proteins and signaling molecules that may be critical for both the migration and aggregation of recently differentiated endocrine cells into islets [9].

INSM1 is involved in a wide variety of transcriptional networks. One functional role of INSM1 is as a repressor in the transcriptional network of the endocrine pancreas to coordinate differentiation into β-cells. INSM1 binds to the promoter region of its gene and is autoregulative, but it also works alongside two other key transcription factors, NeuroD/β2 and Neurogenin3 (Ngn3) [2]. While the transiently expressed Ngn3 induces the expression of the other two transcription factors, INSM1 acts as a repressor for the NeuroD/β2 gene [10]. In this pathway, when the Ngn3 gene is silenced, INSM1 functions as a checkpoint regulator of NeuroD/β2, thus helping coordinate differentiation into β-cells. Similarly, INSM1 is involved in various other transcriptional networks coordinating the differentiation of many other developing neuroendocrine tissues, including sympathoadrenal cells and multiple entero-endocrine cell subtypes: substance P^+^, neurotensin^+^, serotonin^+^, CCK^+^, and PYY^+^ cells [11].

Neuroendocrine tumors (NETs) are relatively rare neoplasms of ‘neuroendocrine’ epithelial cells, which include a variety of endocrine and nervous system cells [12]. NETs can be categorized as high grade to low grade based on their morphologic features. While the latter group typically purports less risk of distant metastases, the former is generally considered much more aggressive with a dismal prognosis. However, it is essential to note that NETs are a heterogeneous group of tumors, and each case may share characteristics of other categories [12]. Therefore, an accurate diagnosis of NETs is of paramount importance for clinical decision-making and personalized treatments.

Aside from the functional relevance of INSM1 as a transcription factor during neuroendocrine differentiation, INSM1 has recently emerged in clinical practice as a diagnostic nuclear biomarker of neuroendocrine differentiation in neoplastic (primarily NETs) tissues and several normal human tissues [13,14] on histology material. However, there are few studies available to address the diagnostic role of INSM-1 in cytologic material. Fine needle aspiration (FNA) is a well-accepted procedure for the initial evaluation of mass lesions, including those with neuroendocrine differentiation. Therefore, it is crucial to utilize reliable neuroendocrine immunomarkers on cytosmears, cell blocks or small core biopsies in order to render a definitive diagnosis. Evaluating the performance of the INSM1 immunostain, including its specificity and efficacy as a nuclear marker on cytology material, is important for a better understanding of its clinical application as a diagnostic immunohistochemical marker. This review will elucidate the clinical application of INSM1 as a nuclear biomarker of various NETs, and briefly discuss its expression in non-neoplastic tissues on cytologic material from cytopathologists’ points of view.

## 2. INSM-1 Detection in Cytologic Material: Pitfalls, Validation and Quality Assurance

INSM1 is detected immunohistochemically in histology and cytology specimens. In cytology, smears, cytospins, liquid-based (LBC) preparations, or cell blocks (CBs) can be used for immunostaining. In practice, laboratories use either one or a combination of cytology preparations, i.e., 60% of European laboratories used a combination of cytology preparations [15]. In detail, CBs were used for immunocytochemistry (ICC) in 38%, cytospins in 22%, LBC preparations in 21%, and smears in 19% alone or in combination with other preparations in 245 surveyed European laboratories that responded to the European Federation of Cytological Societies survey in 2019 [15]. This is a considerable change in comparison to the 2011 survey, when only 20% used CBs and main preparations were conventional smears [16].

A robust survey of 818 United States (US) laboratories showed only 345 respondents used cytology material in immunohistochemistry. 48.8% of laboratories used formalin-fixed paraffin-embedded (FFPE) cell blocks including 25.5% of laboratories used FFPE cell blocks, 21.2% used alcohol-fixed material, post-fixed in formalin, and 2.1% of laboratories used alcohol-fixed material embedded in paraffin without any formalin [17]. There are a wide variety of CB preparation techniques used in the pathology laboratories with plasma-thrombin, agar and collodion bags being considered the most applied [18,19]. Triage of the material is recommended to enhance CB cellular yield [18]. Furthermore, professional societies have issued guidelines for handling cytology specimens for ancillary techniques [20].

The validation process is essential as cytology material is alcohol-fixed or methanol-fixed in the majority of laboratories, which in turn it may lower intensity of immunoreactivity or even it may cause false-negative results.

Importantly, National External Quality Assessment Service for immunohistochemistry of the United Kingdom showed the quality of immunohistochemical results of almost all non-formalin fixatives cell blocks, namely Delaunay-, methanol-, and ethanol-based solutions and the ethanol- and formalin-based CytoRich Red were comparable to those specimens fixed in formalin alone [21]. Thus, local laboratory validation of fixative for immunohistochemistry in cytological preparation is crucial [22]. A total of 44.7% of laboratories out of 311 indicated that the platforms for cytology IHC and positive controls differed for all antibodies in a US laboratory survey [17]. On the other hand, the majority of European surveyed laboratories (96%) performed immunohistochemistry on automated platforms using protocols that were the same as those used for formalin-fixed, paraffin-embedded samples (45%), either optimized (26%) or optimized and validated (29%) for cytology preparations. Furthermore, positive control slides were used in 78% of laboratories and negative control slides in 50% [15]. INSM1 in thyroid medullary carcinoma cytological material was elegantly validated in a multi-institutional international study with various CB preparations (plasma-thrombin, Histogel, Cellient, and in-house method) [23], and collection of visible tissue fragments [24]. Several institutional studies showed INSM-1 reliability in cytological material of neuroendocrine tumors vs the tissues and its superiority in comparison with other neuroendocrine markers [25]. The majority of cytological studies were performed in pulmonary cytological specimens; small cell lung carcinoma expressed INSM1 with 97% sensitivity and 100% specificity [26,27]. INSM1 staining was positive in all 20 paired Cellient CBs and surgical pathology specimens of pulmonary neuroendocrine neoplasms [28]. In another study, matched direct smears and small biopsy samples were concordant in 91% of cases [27]. In a large study including pulmonary small cell carcinomas, large cell carcinomas, and carcinoids, INSM1 sensitivity was 92.3% and specificity was 100% in CBs, along with a 86.2% concordance with surgical specimens [29].

Overall, every pathologist should consider potential sources for errors in each antibody applied, i.e., false-positivity due to cross-reactivity, aberrant and overlapping expression both in application and interpretation of results. Cytological material is suitable if the antibody and detection protocol is validated and appropriate controls are used.

## 3. INSM1 Expression in Head and Neck

In the head and neck area, sinonasal neuroendocrine carcinoma and neuroendocrine carcinomas localized in the hypopharynx, larynx, trachea, and parapharyngeal space represent primary entities, while secondary neuroendocrine neoplasms can be detected in lymph nodes or soft tissue. A systematic study on the expression of INSM1 in head and neck neuroendocrine tumors and carcinomas performed by Rooper et al. [30] showed that also SMARCB1-deficient sinonasal carcinoma, sinonasal adenocarcinoma, alveolar rhabdomyosarcoma, salivary adenocarcinoma, NOS, mucoepidermoid carcinoma, and acinic cell carcinoma expressed INSM1.

HPV-related neuroendocrine carcinomas have been described with small cell, large cell, or combined cell populations, and INSM1 was expressed along with other NE markers [31]. Another study reported INSM1 expression in two cases of HPV-related neuroendocrine carcinoma in cytology series from two different institutions [32].

Paraganglioma is often found in the neck area as a primary tumor or metastasis to the cervical lymph nodes. However, large cytological series of paraganglioma cases did not study INSM1 expression as NE marker in cytological material [33]. Thyroid paraganglioma is supposedly INSM1 positive, but no data is available in the sparse literature on this rare entity [34].

In a multi-institutional series of 49 thyroid medullary carcinomas, cytological specimens (including 29 primary tumors and 20 metastases), and 20 control thyroid gland carcinomas and secondary tumors, 93.75% of medullary carcinomas revealed INSM1 nuclear positivity including all primary tumors showing a sensitivity of 92.3% and a specificity of 100% to discriminate medullary carcinoma from other thyroid malignancies [24]. In addition to medullary carcinoma and its mimickers, INSM1 was successfully applied as a NE marker in C-cell hyperplasia [35].

Malignant thyroid gland teratoma with recurrent *DICER1* hotspot mutations showed patchy INSM1 positivity [36], however a cytological study on this entity did not show any immunoreaction for INSM-1 [37].

A tissue microarray series of 111 cases consisting of normal parathyroid (*n* = 14), primary hyperplasia (*n* = 15), secondary hyperplasia (*n* = 10), tertiary hyperplasia (*n* = 11), adenomas (*n* = 50), atypical adenomas (*n* = 7), and carcinomas (*n* = 4) revealed no INSM1 expression in parathyroid normal tissue, hyperplasia or neoplasms [38].

## 4. INSM1 Expression in Pulmonary NETs

Pulmonary NETs are a subset of pulmonary neoplasms, which contain secretory granules and express neuroendocrine markers. There are four subtypes of pulmonary NETs: small cell lung carcinoma (SCLC), large cell NE carcinoma (LCNEC), atypical carcinoid tumor (ATC), and typical carcinoid tumor (TC) [39]. Since prognosis and management differ significantly based on the subtype of pulmonary neuroendocrine tumors, accurate classification of the pulmonary NETs is critical. Cytomorphological features and clinical behavior are the basic diagnostic criteria, but these characteristics often overlap between different pulmonary NET subtypes. This overlap poses difficulties in the classification of pulmonary NETs. Fortunately, neuroendocrine markers may assist diagnosis by identifying the neuroendocrine lineage of a tumor, serving as a more promising classification strategy. INSM1 has recently emerged as a reliable prognostic immunostain of neuroendocrine differentiation in human lung neoplasms [14]. A study by Doxtader et al. found that INSM1 was 90% sensitive and 100% specific in the detection of pulmonary lung NE neoplasms [28]. These results were confirmed by Viswanathan et al., who also reported an INSM1 sensitivity of 92.3% and specificity of 100% for pulmonary NETs [29]. Multiple studies have affirmed high specificities of INSM1 in SCLCs, up to 100% [27,28]. Sensitivities of INSM1 for LCNEC have ranged from 75% [14] to 91.3% [30]. INSM1 has been found to have high sensitivities in carcinoid tumors, ranging from 98-100% across many studies [14,30,40]. A proof of concept study by Doxtader et al. reported findings from INSM1 staining in 74 specimens and found that INSM1 was positive in 92% (48/52) of primary lung NE neoplasms, including 93% (38/41) of SCLCs, the only case of LCNEC and 90% (9/10) of carcinoid tumors, and negative in all 22 of non-neuroendocrine primary lung tumors including 11 adenocarcinomas, 9 squamous cell carcinomas, 1 mesothelioma, and 1 poorly differentiated non-small cell lung carcinoma, not otherwise specified [28].

### 4.1. INSM1 Expression in SCLCs

SCLC is considered one of the most aggressive malignancies with a dismal prognosis and limited therapeutic options, and it comprises 20% of all lung cancers [41]. INSM1 is expressed almost exclusively in SCLCs.

**Figure 1:**
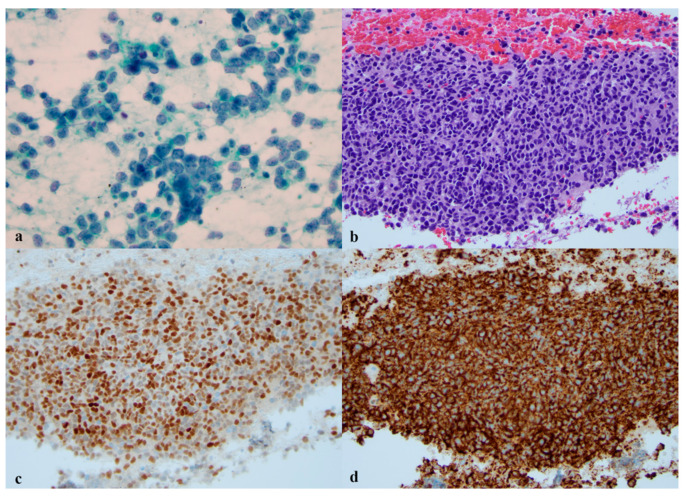
Lung small cell carcinoma. (**a**) A smear shows clusters of loosely cohesive of relatively uniform small cells with high nuclear to cytoplasmic ratio. The chromatin is powdery and the cytoplasm is scant. There is focal nuclear molding and necrosis is seen in the background (×400, Papanicolaou stain). (**b**) A cell block shows a tissue fragment of malignant cells (×200, H and E stain). (**c**) INSM1 highlights neuroendocrine tumor cells, nuclear staining on cell block (×200, IHC). (**d**) CD56 is positive in neuroendocrine tumor cells, cytoplasmic membrane staining on cell block (×200, IHC).

In one study of 32 cases of SCLC, INSM1 was positive in 97% of cases [26]. A large study of 141 NE tumors (78 SCLCs) found similar results, where INSM1 was expressed in 92% of the SCLC cases [42]. INSM1 was able to confirm a diagnosis of SCLC in 9 out of 12 cases, which were all negative for Syn, CgA, and CD56 [42]. This finding is important as it suggests the superiority of INSM1 to the conventional NE markers. Figure 1a–d show an example of SCLC and the application of INSM1.

To date, multiple studies have reported comparisons of INSM1 sensitivity and specificity with CD56, SYP, and CGA in SCLCs. Several studies have documented results showing INSM1′s sensitivity to be largely on par, if not slightly higher, than the sensitivity of SYP [14,28,43]. As compared to CGA’s sensitivity in SCLCs, the consensus in literature thus appears to be that INSM1 is more sensitive. A study by Narayanan et al. found INSM1 to reach sensitivities up to 100% in SCLCs, while CGA comparisons reveal that it underperforms in SCLCs at around 80% [44]. The comparison of INSM1 with CD56 has been more variable. Some studies have determined INSM1 sensitivity to be on par with CD56 [14,26], while others have found INSM1 sensitivity to be lower than CD56 [28]. Further study into the comparisons between traditional NE markers and INSM1 may be useful to confidently reach a consensus on the usefulness of INSM1 as a novel NE marker in SCLCs.

### 4.2. INSM1 Expression and Comparison with Other NE Biomarkers in the Lungs 

Multiple studies have reported comparisons of INSM1 sensitivity and specificity with those of CD56, SYP, and CGA in lung NETs, but there does not yet appear to be a universal consensus on INSM1′s performance. In one study, in the cohort of carcinoid tumors, the sensitivity of INSM1 was lower than all other markers (90% vs. 100% each) [28], while another study found that all markers stained 100% of carcinoid tumors [14]. At the lower end of INSM1′s sensitivity in LCNEC’s (75%), it appears to underperform CD56 (92%) and SYP (88%) while overperforming CGA (46%) [14]. At the upper end of its sensitivity in LCNEC’s (91.3%), INSM1 overperforms a combined panel of traditional markers (78.3%) [30]. In a large study of 345 primary lung neoplasms, INSM1 was found to have a specificity for lung NETs (97%) which is similar to CGA (98%), but greater than CD56 (87%) and SYP (90%) [14].

INSM1 has also been tested with Syntaxin-1 (STX1), a recently described NE marker that is both highly sensitive and specific. In a study that tested both markers in typical carcinoids, atypical carcinoids, SCLCs, and LCNECs, researchers found that though the overall sensitivity of STX1 in these samples was very high (96.6%), INSM1 outperformed the marker (97.7%) [45]. INSM1 also demonstrated its superior sensitivity to classic NE markers, with other markers reporting overall sensitivities of 85.2% (CGA), 85.2% (SYP), and 92.9% (CD56). The findings indicate that a panel of INSM1 and STX1 may be reliable and robust in routine diagnostics of pulmonary NETs [45]. However, more studies are needed before moving to replace traditional neuroendocrine markers with INSM1. In a large cohort of 402 lung tumors, including typical and atypical carcinoids, LCNECs, SCLCs, and thoracic paragangliomas, INSM1 performed at a 76% sensitivity and 99% specificity. All traditional NE markers (SYP, CD56, and CGA) and their combination outperformed in sensitivity (97%) but were lower in specificity (78%) compared with INSM1 [46]. In another cohort of 54 lung NETs, INSM1 performed lower than CD56 (87%), higher than CGA (56%), and lower than SYP (85%) [47].

**Figure 2:**
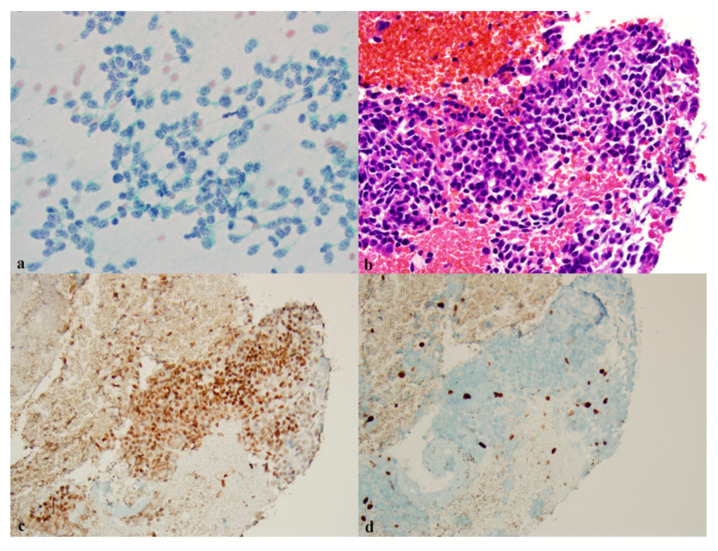
Pulmonary atypical carcinoid. (**a**) Loosely cohesive cells are seen with round to oval nuclei containing coarse chromatin on a smear (×400, Papanicolaou stain). (**b**) Cell block shows neoplastic cells and small foci of necrosis (×200, H and E stain). (**c**) INSM-1 is positive in most neoplastic cells on cell block (×200, IHC). (**d**) KI-67 shows a low proliferation index on cell block (×200, IHC).

Several studies suggest that a panel of NE markers increases the sensitivity for the detection of pulmonary NETs. Viswanathan et al. also reported that the addition of CD56 to INSM1 increased the sensitivity of detecting lung NETs to 100% [29]. Another study found that a panel of CD56, p16, and thyroid transcription factor-1 (TTF1) actually outperformed INSM1 in the diagnosis of SCLC in biopsy samples and cell blocks. The triple marker panel resulted in the correct classification of 100% of SCLC cases (100 samples), while INSM1 was expressed in 81% of cases [48]. Nevertheless, a panel of three markers is more expensive and laborious to perform than the use of a single marker. Figure 2a–d is an example of INSM1 expression in an atypical carcinoid tumor.

Though INSM1 is a diagnostic marker for NETs of the thoracic cavity, there are other non-NET neoplasms of the thorax that may express INSM1. In one study, 7/38 (18%) of lung adenocarcinomas, 2/17 (12%) of lung squamous cell carcinomas, 4/10 (40%) of thymic carcinomas, 4/12 (33%) of adenoid cystic carcinomas, 1/19 (5%) of diffuse large B-cell lymphomas, 4/11 (36%) of alveolar rhabdomyosarcomas, and 4/23 (17%) of Ewing sarcomas were positive for INSM1. No synovial sarcomas or desmoplastic small round cell tumors were positive for INSM1. Therefore, it is of paramount importance to pay close attention to cytomorphologic features and clinical presentation of thoracic neoplasms in order to avoid diagnostic pitfalls and implement ancillary studies for further characterization of neoplastic cells mimicking NETs [49]. The lower end of INSM1′s sensitivity and specificity has been found to be 81.5% and 82.7%, respectively; however, INSM1 positivity higher than the minimum diagnostic threshold was observed in rare thoracic tumors that have a morphologic overlap with SCLCs [49]. Table 1 compares the findings of several studies. Overall, INSM1 shows high sensitivity and specificity for the diagnosis of lung NETs.

## 5. INSM1 Expression in Pancreatic NETs

Pancreatic NETs are a subset of pancreatic neoplasms with neuroendocrine differentiation. They can pose diagnostic challenges due to variable clinical presentations, heterogenous cytomorphology, and variable grading. They may clinically present as a solid or cystic pancreatic mass [50]. Their cytomorphology is variable including oncocytic, hepatoid, lipid-rich, plasmacytoid, ductuloinsular, pleomorphic, and paraganglioma-like [51]. Pancreatic NETs are rarely associated with psammoma bodies and stromal amyloid deposition [52]. A study found that the detection rates of INSM1, CG, and SYN were 100%, 95%, and 100%, respectively [53].

Moreover, pancreatic NETs can present as pure tumor or combined with other non-NETs, such as adenocarcinoma. The aforementioned markers (SYP, CGA, and INSM1) were evaluated with both pure primary pancreatic NET and mixed adenoneuroendocrine carcinoma (MiNEN, which contains both NET and carcinoma components). This revealed that INSM1 was strongly positive (100%) for the pure NET and the NET component of MiNEN, and was negative for the adenocarcinoma component (as seen with other GI-tract pure carcinomas). The other conventional NE biomarkers are not as sensitive for the pure NET, and CGA failed to stain NET component of a MiNEN NET sample, suggesting that INSM1 could be a more sensitive biomarker for diagnosing pure NETs or identifying NET components in mixed cases [54]. In fact, in an analysis of cell block samples, INSM1 displayed 100% sensitivity, specificity, positive predictive value (PPV), and negative predictive value (NPV) for differentiating NET from non-NET pancreatic tumors, as SPN (solid pseudopapillary neoplasm), ACC (acinar cell carcinoma), and PDAC (pancreatic ductal adenocarcinoma) samples were always INSM1 negative (unlike SYP which was positive for SPN), making it a useful marker for excluding possible non-NET diagnoses [54,55]. Since it has a high NPV for PDAC, it can be useful for excluding exocrine pancreatic tumors in a differential diagnosis.

The pancreatic NET grades have shown to be inversely correlated with INSM1 nuclear expression. In a study, INSM1 expression was given an H-score (H score considered the level of expression (0-3) and the percentage of cells stained and then weighed for a score between 0 and 300) in 55 pancreatic NETs with variable tumor gradings. The H-score was approximately 30 for G3 pancreatic NETs, 229 for G1 and 266 for G2 tumors [55]. The results for INSM1 expression was identical for both pancreatic NETs on cell block and surgical resection. This corroborates the finding subsequently discussed for gastroenteric NET’s where Grade 3 NET’s exhibited similar findings with INSM1 staining. Solid pseudopapillary neoplasms (SPN) can sometimes create difficulty in the diagnosis of NET due to similarities in presentation. Conveniently in the case of SPN, only a weak, cytoplasmic INSM1 staining in focal areas was observed, which is interpreted as a negative result [54,55]. Other markers such as SYP were positive in 40% of the SPN cases [54,56]. This observation suggests that INSM1 is a useful biomarker for excluding SPN. Certain NE biomarkers, including SYP for example, are known to be expressed in entities, such as solid pseudopapillary neoplasm (SPN) of the breast. The fact that conventional NE markers are non-specific led to a search for an alternative NE marker, such as INSM1 [25]. 

## 6. INSM1 Expression in Gastroenteric NETs

The category of gastroenteric NETs, including gastric, intestinal, rectal, and appendiceal, is heterogeneous and may be quite clinically aggressive due to the risk associated with their location, higher grade, and high mitotic index. Current conventional NE biomarkers used in gastroenteric-NET diagnoses are SYP and CGA immunostains. INSM1 has been proven to be useful in identifying NETs with its strong and diffuse nuclear staining. Rectal NETs are largely negative for CGA; however, they are reactive to INSM1 (such as SYP) in 100% of the cases [57]. In primary NETs of the intestines and stomach, nuclear expression of INSM1 was shown to be on par with SYP and CGA staining, which was in 100 % or nearly 100% of the cases. Furthermore, INSM1 exhibited high reactivity, staining between 85% to 92% (on average) of the total cells for G1, G2, and G3 primary NETs. However, metastatic G3 NETs were shown to be reactive for SYP and CGA in a higher percentage of cases than INSM1 [55,56].

Non-NETs like carcinomas, such as colorectal adenocarcinoma, almost always were negative for INSM1. However, other NE markers such as SYP were immunoreactive in approximately 50% of the colorectal adenocarcinoma cases [54,56].

A study on 110 gastrointestinal NETs showed that INSM1 was positive in 94.1% of gastric (16/17), 72.2% of small bowel (13/18), 81.0% of colonic (17/21), and 72.2% of appendiceal tumors (26/36). INSM1 was positive in 82.9% of well-differentiated neuroendocrine tumors (58/70), 85.0% of poorly differentiated neuroendocrine carcinomas (17/20), 72.7% of low-grade goblet cell adenocarcinomas (8/11) (grade 1), and 66.7% of high-grade goblet cell adenocarcinomas (6/9) (grade 2/3). INSM1 was slightly less sensitive (80.9%) for detection of gastroenteric neuroendocrine neoplasms than that of SYP (99.1%), CGA (88.6%), and CD56 (95.3%) while it was more specific (95.7% vs 86.0%, 87.3%, and 86.0%, respectively) [58]. Due to the non-specific background noise and spotted membranous and cytoplasmic staining seen with SYP and CGA, INSM1 seems to be a superior NE biomarker for gastroenteric NETs. INSM1 can detect primary NETs by their strong and diffuse nuclear expression, in particular in cases with unusual cytomorphologic features. Specifically, it can be useful as a supplemental stain when conventional staining is difficult to interpret or if INSM1′s NPV (negative predictive value) with non-NETs are needed to exclude a diagnosis [55]. Non-NETs of GI tract, such as cholangiocarcinomas, and adenocarcinomas of the colon, can sometimes be weakly and focally positive for INSM1 [53].

## 7. INSM1 Expression in Breast Carcinomas with Neuroendocrine Differentiation

Primary NETs of the breast are rare entities and are often underrecognized. NETs of the breast occur most commonly in postmenopausal women [59]. The emergence of INSM1 as a more sensitive “second-generation” biomarker for neuroendocrine differentiation in the breast holds promising prospects for improved diagnosis of breast NETs, which are difficult to distinguish from other types of breast carcinomas based on histologic features alone. To date, few studies have analyzed the usefulness of INSM1 in detecting neuroendocrine differentiation of invasive breast carcinomas. Moreover, few studies have compared INSM1 to other types of neuroendocrine markers in the context of neuroendocrine breast carcinomas (NEBCs). As such, the effectiveness and comparative diagnostic value of INSM1 in diagnosing NEBCs is a topic that necessitates further studies. Breast cancer occasionally is focally and weakly positive for INSM-1 [53]. Neuroendocrine differentiation can also be associated with invasive breast carcinomas in up to 30% of the cases. Neuroendocrine carcinoma of the breast is defined as a subset of invasive mammary carcinoma in which neuroendocrine deafferentation is expressed in greater than 50% of the tumor cells [60]. Morphologically, NETs of the breast are categorized into well differentiated NETs, poorly differentiated NETs or small cell carcinoma, and invasive breast carcinoma with neuroendocrine differentiation [61]. The latter is most commonly associated with solid papillary and mucinous carcinoma [61]. Well differentiated NETs, and poorly differentiated NETs or small cell carcinoma of the breast are identical to their pulmonary counterparts [61].

Identifying neuroendocrine differentiation in mammary neoplasms can be helpful in diagnosis of some special types of breast carcinomas, such as solid papillary variant of NECB. However, diagnosis can be a challenge, as these special type of breast carcinomas do not consistently display histologic features of neuroendocrine differentiation [60]. Diagnosis of neuroendocrine differentiation is based on the expression of neuroendocrine markers. Neuroendocrine markers that have been the most commonly utilized for the diagnosis of mammary NETs are CgA, SYP, NSE (neuron-specific enolase), and CD56.

More recently, INSM1 has emerged as sensitive biomarker for the detection of neuroendocrine differentiation in breast neoplasms. A study by Roy et al. found that INSM1 was diffusely expressed in five out of seven breast carcinoma cases with neuroendocrine differentiation (similar to CgA and CD56 expression) while SYP was expressed in six of the seven cases [59]. In a study on three breast neoplasms from three Japanese female patients, ages 42, 58, and 64, Kawasaki and Kaira found that INSM1 was expressed strongly and diffusely in all three cases (1 NET, 1 mucinous carcinoma, and 1 neuroendocrine carcinoma in situ), while CgA and SYP were negative in all three cases [62]. A study by Seijnhaeve et al. compared SYP and CgA with INSM1 by using a validation cohort of 22 mammary neoplasms (8 solid papillary carcinomas, 6 mucinous carcinomas, 3 invasive carcinomas, 3 encapsulated papillary carcinomas (SPC), and 2 spindle cell variants of ductal carcinoma in situ cases) with or without neuroendocrine differentiation. Of the 22 cases, 5/22 were SYP+/CgA+/INSM1+, 1/22 was SYN+/CgA+/INSM1-, 8/22 were SYN+/CgA-/INSM1+, and 3/22 were SYN-/CgA-/INSM1+, while 5/22 cases were negative for all three markers. Overall, INSM1 was expressed in 16/22 cases, which was higher than those for SYN (14/22) and CgA (6/22) [63]. INSM1 expression was found in all solid papillary carcinoma cases (5% to 99% nuclear expression), while mucinous carcinomas showed minimal nuclear expression (1% to 5%) [63].

Solid papillary carcinoma is a subtype of breast carcinoma showing neuroendocrine differentiation in at least 50% of cases [64,65]. Kudo et al. evaluated INSM1 performance using 19 SPC cases including 11/19 with neuroendocrine differentiation and 8/11 without neuroendocrine differentiation. INSM1 was expressed in 9/11 cases with neuroendocrine differentiation and 2/8 SPC without neuroendocrine differentiation [66]. The intensity of the INSM1 stain was significantly higher among SPC with neuroendocrine differentiation than in non-neuroendocrine SPCs [66].

Though there is no clear consensus on the prognosis of these tumors [67], most studies suggest a poor outcome. Neuroendocrine differentiation is considered as an independent adverse prognostic factor for overall survival and disease specific survival [61]. NETs of the breast show a remarkably higher rate of local and distant recurrence than invasive ductal carcinoma (not otherwise specified) [61]. Therefore, an accurate diagnosis of NETs of the breast may play a critical role in clinical decision-making and patients’ management, although currently they are treated similarly to other invasive breast carcinomas.

## 8. INSM1 Expression in Genitourinary Tumors

NETs can present in a variety of locations within the reproductive and urinary tracts. Neuroendocrine carcinomas (NEC) of the female genital tract are rare, aggressive malignant neoplasms. Location seems to determine the effectiveness of INSM1 staining for neuroendocrine carcinomas (NEC) in the female genital tract. In a study on 49 gynecologic NECs (4 vagina, 39 cervix, 5 cases of endometrium, and 1 ovary), INSM1 expression was more diffuse and intense compared to SYN, CgA, and CD56 for cervical small cell NECs but not for large cell NECs. However, for endometrial NECs, the other NE markers performed better, as INSM1 was only focally and weakly expressed [68]. For high grade neuroendocrine carcinomas of the uterine cervix, INSM1 was more sensitive (92% of cases) than SYP and CgA (both reactive up to 86% of cases), and its expression was also more clearly interpretable due to its nuclear staining [68].

In the male reproductive system, small cell carcinomas of the prostate are known for their aggressive behavior and poor prognosis. Therefore, an accurate diagnosis is crucial for proper patient management. For prostate small cell carcinoma, INSM1 has been shown to be more sensitive (92.3%, 12/13) compared to SYP (84.6%, 11/13) and CgA (53.8%, 7/13), making it the superior NE marker for prostate small cell carcinoma. Moreover, it was quite specific, since only 3.4% of benign prostate tissues and 4.0% of prostate adenocarcinomas stained positive for INSM1 [69].

A study on 13 cases of clear cell renal cell carcinoma (CCRC) with Paneth-like cells concluded that none of the cases expressed NE markers, including INSM1 [70].

Another study evaluated INSM1 expression in 39 high grade NE carcinomas (4 in kidney, 28 in urinary bladder, and 7 in prostate); 31 small cell carcinomas (SmCCs), 6 large cell neuroendocrine carcinomas (LCNECs), 2 mixed SmCC-LCNECs) of the genitourinary tract and it compared it to SYN, CgA, and CD56. INSM1 showed similar sensitivity (93.9%) to SYN (93.9%), CgA (87.8%), and CD56 (87.8%) in all 33 small cell carcinomas or carcinomas with small cell carcinoma components. INSM1 expression (52%) was similar to that of chromogranin (49%) and CD56 (52%), but lower than that of synaptophysin (87%) (p < 0.0001). In eight large cell NE carcinomas or carcinomas with large cell components, INSM1 showed similar sensitivity (6/8) to SNY (7/8), CgA (6/8) and CD56 (6/8). Overall, INSM1 was more sensitive for the detection of small cell carcinomas than for large cell NE carcinoma (93.9% vs. 62.5% (6/8), *p* = 0.015) of genitourinary tract. INSM1 was also highly specific (97.4%) for the detection of genitourinary neuroendocrine carcinomas [71].

## 9. INSM1 Expression in Skin and Skin Appendages

**Figure 3:**
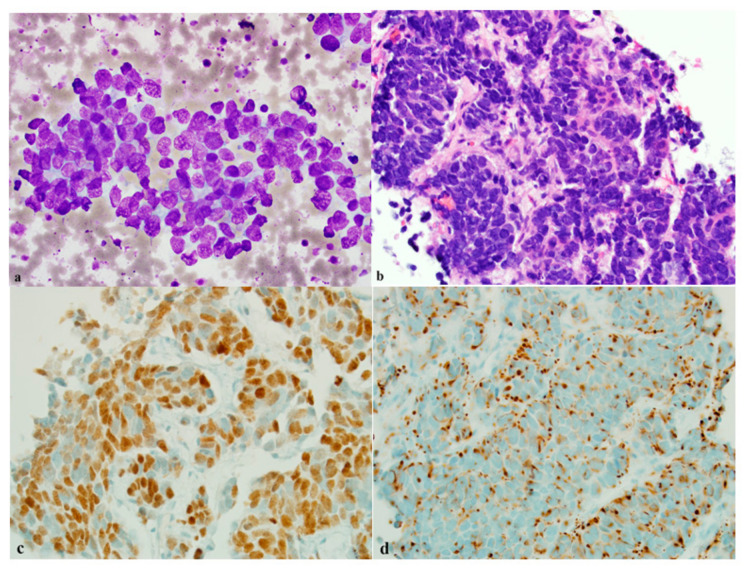
Merkel cell carcinoma. (**a**) Malignant cells are characterized by round to oval nuclei, high nuclear to cytoplasmic ratio, scant cytoplasm, and focal nuclear overlap on a smear (×400, Diff-Qiuk stain). (**b**) A small core biopsy shows nests of malignant cells (×200 H and E). (**c**) INSM1 stains nuclei of malignant cells strongly and diffusely on a core biopsy (×200, IHC). (**d**) CK20 is positive, perinuclear cytoplasmic staining on a core biopsy (×200, IHC).

Merkel cell carcinoma (MCC) is a neuroendocrine carcinoma of the skin. MCC diagnosis can be impeded by its morphologic overlap with other neoplasms, such as basal cell carcinomas and squamous cell carcinomas, especially when presenting with atypical immunophenotypes [72]. Accurately diagnosing MCC can improve treatment outcomes for this cancer that is known particularly for its aggressive behavior. A study of 56 MCC (47 primary tumors, 9 nodal metastases) and 50 control cases (basal cell carcinomas, basaloid squamous cell carcinomas, Bowen disease, sebaceous neoplasms, melanoma, and B-cell lymphomas, and 28 lymph node control cases that included metastatic neuroendocrine neoplasms, melanomas, squamous cell carcinomas, lymphomas, and adenocarcinomas), INSM1 was expressed in all 56 cases including primary and secondary tumors, while synaptophysin, CK20, and chromogranin were expressed in 96%, 92%, and 32% of MCC cases, respectively [72]. All control cases with the exception of the lymph nodes involved with NE tumors (one carcinoid of small bowel, two small cell carcinoma, and pancreatic NET primary) were negative for INSM1. INSM1 was found to be a very sensitive biomarker for the differential diagnosis of MCC from other non-MCC cases, while it cannot differentiate MCC from other NE carcinomas [72,73,74]. Figure 3a–d show an example of the application of INSM1 in MCC.

Another study of 24 MCC cases revealed that INSM1 was more sensitive (100%) than the other NE markers: SYP (96%), CGA (92%), and CD56 (79%). The INSM1 expression was also strong and diffuse; for instance 21/ 24 cases had positively stained more than 75% of the cells for INSM1, which was more diffuse and more intense than the other NE markers [73]. INSM1 was not expressed in normal cutaneous cells like melanocytes and endothelial cells [72,73,74]. The only non-neoplastic cells that did stain positive were Merkel cells in hair follicles or in the epidermis [73].

INSM1 is a sensitive NE marker for MCC diagnosis; however, it is incapable of differentiating MCC from metastatic neuroendocrine carcinomas of an extracutaneous origin [72]. Therefore, including INSM1 as part of a larger panel of immunostains can be helpful in the diagnosis of MCC. INSM1 positivity was more intense than that of other NE markers in the hidrocystoma-like component of cystic endocrine mucin-producing sweat gland carcinoma [75]. Squamous cell carcinoma is sometimes focally and weakly positive for INSM-1 [53].

## 10. INSM1 Expression in Soft Tissue Tumors

Solitary fibrous tumors (SFT) are mesenchymal neoplasms of soft tissue (commonly observed in the pleura). Cases of SFT were investigated to see if INSM1 could prove to be helpful in its diagnosis. However, it seems that other markers perform superiorly with SFT. A study on 28 SFT cases found that INSM1 was reactive in 21% (6/28) of the cases, which was slightly superior than desmin (14.3%, 4/28) and p16 (17.9%, 5/28), but HTER (46%,13/28) and STAT6 (100%, 28/28) were more helpful immunostains [76]. Differential diagnosis of sarcomas can also be supplemented by the use of the INSM1 marker. Specifically, when differentiating extraskeletal myxoid chondrosarcoma (EMC) from other mesenchymal tumors, INSM1 shows 90% positive staining with EMC but was negative with 94% of the other mesenchymal tumor samples of various cell types [77]. Another study that focused on a variety of sarcomas found that some angiosarcomas (26%, 24/94, mostly diffusely staining positive), a few desmoplastic small round cell tumors (11%, 7/62, weak to strong staining), and rarely some synovial sarcomas (4%, 3/76. moderate to strong staining) were positive for INSM1, while the other sarcomas including Ewing sarcoma (0/57), clear cell sarcoma (0/14), soft tissue leiomyosarcoma (0/59), uterine leiomyosarcoma (0/65), alveolar soft part sarcoma (0/29), epithelioid sarcoma (0/30), and undifferentiated pleomorphic sarcoma (0/100) were negative [78].

## 11. INSM1 Expression in Non-Neuroendocrine Neoplastic and Non-Neoplastic Cells

**Figure 4:**
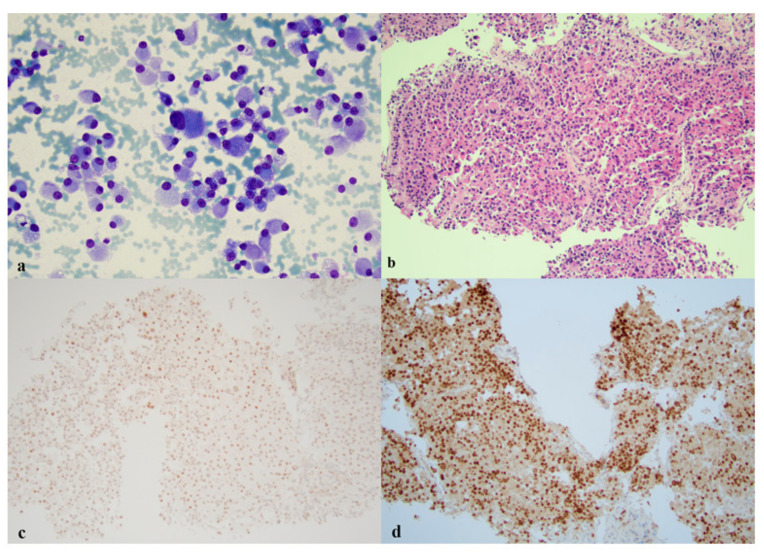
Adrenal cortical carcinoma. (**a**) The malignant cells are plasmacytoid and characterized by abundant cytoplasm and eccentric nuclei on a smear (×400, Diff-Quik). (**b**) A small core biopsy comprised of malignant cells with abundant eosinophilic cytoplasm (×200, H and E). (**c**) INSM1 shows nuclear staining on small core biopsy (×200, IHC). (**d**) SF1 demonstrates nuclear staining on small core biopsy (×200, IHC).

It is important to be aware of non-neuroendocrine neoplastic and non-neoplastic entities, which may stain positive for INSM-1 and result in false-positive diagnoses. Adrenocortical carcinoma cells were also found to express INSM1, but more data is likely necessary before conclusively determining that adrenocortical carcinoma consistently stains positive for INSM1 [25,53]. Figure 4a–d shows the application of INSM1 in adrenocortical carcinoma. Awareness of its positive staining can help prevent misdiagnosing adrenocortical carcinoma as a metastasized neuroendocrine carcinoma or as an extremely rare primary small cell neuroendocrine carcinoma of the adrenal gland [79].

INSM-1 expression in normal tissues may cause diagnostic pitfalls. Pancreatic islet cells and benign adrenal gland cells are notable examples of normal tissues expressing INSM-1 [25]. These cytology specimens should be interpreted carefully. Islet cell hyperplasia in a background of chronic pancreatitis can be misinterpreted as pancreatic NET and INSM-1 can facilitate a misdiagnosis. This is seen in the case of benign adrenal gland cells aspirated and stained with INSM-1. Furthermore, these diagnoses can become even more difficult when non-neoplastic cells in various locations stain positive for INSM1, as seen in a few common locations. For instance, It is useful to be aware that Merkel cells are immunoreactive for INSM-1 in epidermis and hair follicles when assessing potential cutaneous neoplasms [73]. Pancreatic specimens may pose diagnostic challenges as well, due to nuclear expression of INSM1 in islet cells (pancreatic neuroendocrine cells) [25]. In addition, normal cortical adrenal cells and also a very small percentage of prostate cells are positive for INSM1 [25,26,27,28,29,30,31,32,33,34,35,36,37,38,39,40,41,42,43,44,45,46,47,48,49,50,51,52,53,54,55,56,57,58,59].

INSM1 may also be useful in predicting the prognosis of SCLCs, as tumors with low INSM1 expression have been observed to have a “significantly better prognosis” than cases with high INSM1 expression [42]. Those with high expression of INSM1 have been associated with poorer overall survival and recurrence-free survival [80].

## 12. Conclusions

Overall, the efficacy of insulinoma-associated protein 1 (INSM1) as an immunohistochemical marker for neuroendocrine neoplasms has been supported by various clinical studies demonstrating its high sensitivity and even higher specificity for detecting NETs of various sites.

In the head and neck area, sinonasal neuroendocrine carcinoma, neuroendocrine carcinomas localized in the hypopharynx, larynx, trachea and parapharyngeal space, as well as HPV-related neuroendocrine carcinomas, paraganglioma, and medullary thyroid carcinoma were shown to be immunoreactive for INSM1. Thyroid gland teratoma with recurrent *DICER1* hotspot mutations showed patchy staining for INSM1. Parathyroid and follicular cells were negative for INSM1, while thyroid C cells expressed INSM-1. 

INSM1 has recently emerged as a reliable immunostain of NE differentiation in lung neoplasms as well. In particular, INSM1 has shown to be a highly sensitive and specific NE biomarker in identifying SCLCs, and recent comparisons between INSM1 and conventional NE markers indicate INSM1′s superiority over traditional markers for SCLCs. Regarding other lung NETs, multiple studies have compared INSM1 sensitivity and specificity to existing markers, but a universal consensus on the immunostain’s performance has yet to be reached. INSM1 tends to show high sensitivities overall but appears to underperform in comparison to CD56 and SYP in certain lung NETs such as carcinoid tumors and LCNECs. Overall, INSM1 presents itself as a reliable immunostain for the diagnosis of SCLCs and other lung NETs, but more studies are necessary before it can replace traditional NE markers.

In pancreatic NETs, INSM1 is more sensitive and specific than conventional markers in differentiating NETs from non-NET tumors. Interestingly, INSM1 can be useful for MiNEN (mixed component tumors) in identifying the NE components due to its high specificity for NE differentiation. In identifying gastroenteric NETs, INSM1 has been proven to show strong diffuse staining equal to or better than conventional markers, such as CGA and SYP. However, INSM-1 utility has its own limitation in G3 pancreatic NETs and G3 metastatic gastroenteric NETs.

Most importantly, non-NETs remain negative for INSM1 even in challenging cases, such as solid pseudopapillary neoplasm (SPN), which can be positive for SYP.

NE carcinomas of the breast (NEBCs) showed diffuse INSM1 staining. INSM1 is more sensitive than the conventional markers CGA and SYP, but may be less specific than CGA. INSM1 also proved useful in distinguishing NE forms of solid papillary carcinomas (SPC) from non-neuroendocrine SPC.

Overall, in genitourinary neoplasms, INSM1 was more sensitive to small cell NEC compared to large cell NECs. Regarding Merkel cell carcinoma, INSM1 has been shown to stain more diffusely and intensely than markers such as SYP, CGA, and CD56. However, it is not able to differentiate MCC from metastatic NE carcinomas originating from extracutaneous locations.

INSM1 was not shown to be useful for solitary fibrous tumor (SFT) diagnosis when compared to HTER and STAT6.

A variety of non-NE neoplastic cells are positive for INSM1; thus, it is important to be aware of them to prevent misdiagnoses. These include adrenocortical carcinoma, angiosarcoma, desmoplastic small round cell tumor, synovial sarcoma, and clear cell renal cell carcinoma. Non-neoplastic cells, such as epidermal and hair follicular Merkel cells, pancreatic islet cells, benign adrenal gland cells, and prostate cells, have also stained positive for INSM1.

INSM1 is a neuroendocrine marker that offers ease of interpretation in cytology material due to its nuclear expression. Its specificity for NE differentiation promises important value as an additional useful tool in the diagnosis of NETs that either lack or have equivocal expression of “traditional” NE markers. The wide applicability of INSM1 is evident from these studies, and it calls for further meta analyses of the data for better characterization of this NE immunostain. 

## Figures and Tables

**Table 1:** The summary of immunohistochemical expression of INSM1, synaptophysin, chromogranin, and CD56 in pulmonary neuroendocrine tumors.

	Source	INSM1 %, n/N	SYN %, n/N	CgA %, n/N	CD56 %, n/N
**SCLC**	Doxtader et al.	93 (38/41)	93 (37/40)	35 (14/40)	100 (40/40)
	Viswanathan et al.	89(8/9)	78 (7/9)	22 (2/9)	100 (9/9)
	Rodriguez et al.	97 (31/32)	82 (14/17)	62 (10/16)	96 (22/23)
	Narka et al.	97 (36/37)	96 (27/28)	100 (18/18)	100 (6/6)
	Sakakibara et al.	92 (72/78)	55 (43/78)	48 (37/78)	81 (63/78)
	Mukhopadhyay et al.	98 (63/64)	100 (64/64)	83 (53/64)	95 (61/64)
**LGNEC**	Doxtader et al.	100 (1/1)	100 (1/1)	100 (1/1)	100 (1/1)
	Viswanathan et al.	75 (6/8)	63 (5/8)	25 (2/8)	100 (8/8)
	Sakakibara et al.	68 (30/44)	57 (25/44)	44 (18/440	84 (37/44)
	Mukhopadhyay et al.	75 (18/24)	88 (21/24)	46 (11/24)	92 (22/24)
**Carcinoid, Typical and Atypical**	Doxtader et al.	90 (9/10)	100 (10/10)	100 (10/10)	90 (9/10)
	Viswanathan et al.	100 (22/22)	100 (22/22)	100 (22/22)	95(21/22)
	Sakakibara et al.	95 (18/19)	100 (19/19)	100 (19/19)	100 (19/19)
	Mukhopadhyay et al.	98 (63/64)	100 (64/64)	100 (61/61)	100 (58/58)

## Data Availability

Data will be available upon the request.
